# Our Trip to the Medical Congress

**Published:** 1887-10

**Authors:** 


### OUR TRIP TO THE MEDICAL CONGRESS.

I am glad to know that on this beautiful earth of ours there is always an abundance of room for every good and noble work that the heart of man can conceive or his brain execute. We talk sometimes of crowded cities, crowded professions, and a surplus of workmen in many fields of labor, but thank God no one can say there is too much good work done in the world, or too many cheering, sympathetic words uttered to inspire and invigorate the hearts and the action of our fellow men.

Feeling thus, we propose to give our readers our first impressions of the great International Medical Congress, lately convened at Washington, as we sat in the aisle of the large Opera House of that city, as well as many of our thoughts, feelings and conversations during the session of that grand occasion. And first, as I looked over the vast array of medical talent thus assembled, I inwardly exclaimed that in all the history of the medical fraternity, this is to me the biggest of "red letter days." All around me I saw a large number of professional brethren assembled here from all parts of Christendom. They were men who nobly represented the God-like profession of medicine; whose names are illustrious upon two continents, and upon whose heads benedictions, warm from the throbbing heart of suffering humanity, have been often pronounced.

As I sat there in that august assembly, my heart grew big within me, and swelled with joy and pride to feel that I, however unworthy, am honored by being one of the links in the golden bond of fraternal will and good fellowship, and that I am an humble exponent of that noblest of all arts—the healing of the sick, and restorer of joy to anxious loving hearts.

It is a truth to be noted and emphasized, that men of any reputable profession, who exalt that profession, never fail to receive due honor from their friends and the public. Sometimes it is slow in coming, and a man with a noble, aspiring spirit, treads a long and dreary way before the flowers begin to spring up at his feet; sometimes death comes to him before he has received his earthly reward, but the laurels are eventually wreathed about his name, and his life, though crownless here, has made the world better for having been lived out among his fellow men.

I was never so much impressed with the cosmopolitan nature of medicine as I was on this great occasion. I never had my heart to so throb and thrill with the magnetic force of the world-wide brotherhood of humanity. How true it is—and the truth is most welcome to me—that whether from over the seas, from the east, west, north or southern points of the compass, we are brethren in that great fraternal family, with one noble purpose in humanity, with one sky above us, and one God over all.

Here was represented England, a land ablaze with illustrious names in sience art, religion, literature, medicine, statesmanship and military glory—she will ever be to us as a revered and honored parent; may it continue to be said, that upon her vast boundaries the sun never sets, and her morning drum-beat circles the globe. And here, too, was France, the home of the patriot LaFayette, she also is peculiarly dear to the people of these United States. Her generous sympathies and aid, as from one friendly nation to another, are not only matters of history, but are cherished memories transmitted to our children through successive generations. Her investigations and triumphs in medical science have laid, not only the Profession, but the people of all nations under obligations to the land of Devaux, Velpeau, and Lavoesier. Great in national prestige, brave, buoyant, enthused with hope and energy under all disasters, may the star of its young republic grow brighter and brighter as the cycles wane, and Bartholdi's magnificent Statue of Liberty, planted on American shores, gives light to the nations of the earth. To the brethren of Teutonic blood, I could only repeat heart-felt words of fraternity and proud recognition. The profession feels most profoundly its indebtedness to the genius of medicine in that land which has so often lifted its standard out of the quagmires of error, and has brought the light of certainty out of the darkness of experiment, thus giving the medical ranks courage and strength to meet the approaches of insidious disease. No country of our cosmopolitan brotherhood has furnished names of richer lustrue, and notably among them, that of the lamented Von Liebig, who lately left to the profession and the scientific world the most valued legacy of medical chemistry with which it has ever been endowed. When Italy produced a Mondini, who rescued from obscurity the wonderful mysteries of anatomy, regions of incalculable value were opened to modern medicine and thus all lands and all nations have brought their contributions of medical acumen to enrich the profession. Like a reflex action, they have given and received until the tide has flowed back and forth to the uttermost parts of the earth, and the brethren who were welcomed from all the great powers of the continent of Europe and some of the beautiful islands of the Pacific, clasped hands in a fraternal union that is one and indissoluble.

More than twenty years ago, when the Southern section of the United States had taken up arms against the North in defence of its homes and constitutional liberty, and even brothers, ties of blood, were arrayed against each other on the battle-field, the bond of the medical fraternity between the two sections remained unbroken. States may disagree and nations may war with each other, but the *Parliament of Medicine* cannot be convened nor dissolved by any political mandate; but here from every land and nation they came together to receive the greeting and to enjoy the welcome of the American profession.

Whenever the ranks of the profession are doing service upon their own peculiar field of usefulness and honor, they owe allegiance to but one Great Commander, the God of Nature and Supreme Revealer of the Universe.

We believe that members of the medical profession, the world over, are conceded to be patriots, though their calling exempts them from any prominece in the political arena. Hence, if they do no special good in politics, they are on the safe side in doing no harm, a negative virture which is better than a positive evil. As the doctors of the North, and we doctors of the South never separated, but were, and still are fraternally united, we can more readily feel that we are again truly citizens of one common country, and brethren of one family circle, unbroken by discord and dissensions.

After the civil war was over the South accepted the situation pretty much as did a boy who was once nagged into a fight by a schoolmate much larger and and stronger than himself. The latter, though, was full of true grit and fighting fire, and so it was a close drawn battle until finally the smaller boy was compelled to cry "enough." He then rose up and shook himself and said: "I'll own that you licked me that time because you had the advantage all through, but you can't deny that you got pretty badly hurt yourself. Now, if you will do the square thing by me hereafter here's my hand and we will be friends as before."

I am glad to know that all true patriots, North and South, are ready to do justice to each section, and in national union interchange fraternal love and sympathy. Some of the stalwart partisans at the North have said that until the South confesses that the war against the North was wrong there can be no real fraternization; but the South feels that when the North pays its part for the liberated slaves, the renewed bond of union between the sctions will be riveted no more to be broken.

This is but simple justice, as Horace Greely and other representative men of the North have admitted. For some wise purpose the Supreme Ruler of all nations saw it was best that the United States should not be dismembered by the civil war.

The South never made war upon the Union, nor the American Constitution, but after it failed to secure honorable terms of peace, it took up arms against a relentless partisanship, and in defence of its homes, property and constitutional rights. When the first blow was struck, I do not believe that there was a Southerner who did not regret the necessity of the action.

The beautiful Stars and Stripes, that had led Southern men with their Northern brethren to many blood-bought victories in generations past, was lowered now, with sighs and tears to the departed peace and harmony of the Republic. When the first gun was fired at Sumpter, in all this broad land there were no more loyal hearts to the spirit and letter of the American Constitution than the descendents of Washington, Randolph, Clay, Calhoun, and a host of immortal Southern heroes who had so largely shaped the destinies of this nation, on the battle field or in the national councils.

During the civil war and its bitter aftermath, every brave, heroic, and magnanimous word or deed that was uttered or committed by Northern men was, and is now, recognized by the South with national pride and treasured among its memoirs; and so I believe that every true patriot at the North will do justice to the honest convictions, the heroism and lofty bearing of the South through all its long and firery ordeal of war and reconstruction of the Southern States. But I rejoice that all these unhappy national memories are now to be relegated to the past for all time, and that men of the North and men of the South can set here to-day and feel that in professional fraternity their hearts are one, and in national bonds both sections can again say we are brothers and citizens of one common country, with the soul-thrilling watchword, "the union of these States are one and inseparable."

Corrupt political malcontents may continue to stir the seething cauldron of hate and disunion, but the true men of both the North and the South will stand with united hearts and hands in a solid phalanx to promote the highest good, and the peace, happiness and prosperity of the whole country, and to bring to rich fruition the grand prophecies of America's mighty Republic. If every American would resolve to do this, our Government would become fixed upon a granite basis of safety in union, and as the blazing comet illumines the high arch of heaven, this great continent would be lit up with patriotic fire from North to South, from East to West, and the stars and stripes of our glorious old flag would continue to float over the land of the brave until

> "Wrapt in flames the realms of ether glow,
> And heaven's last thunders shake the world below."

To establish this firm and fraternal unionism the medical profession, North and South, can wield a great influence. They can greatly aid in restoring harmony between the sections, and the former strength and beauty of that grand tree of liberty our forefathers planted in this beautiful and historic Capital City and that was baptized by the blood of both Northern and Southern heroes.

These professional brethren can exhort the North and the South to say to each other, "with all thy faults I love thee still," and hand in hand we will grandly move on to accomplish the high destinies of our beloved country; then, when time with us shall be no more, still hand in hand may we enter through the gates of pearl into the golden city of God, and live forever there in one common country as in earth, and as one family in the eternal mansions in the skies.

And these brethren here from every portion of the globe, are not we all citizens of one common country—the great sublime and world-wide country that embraces the four quarters of the earth, and is called by the grand name of humanity? Wherever men labor, love, hope, suffer and pray there is our country. Men who try to relieve the sorrows of earth and who do good and noble deeds, they are our brothers; and men who do homage to wise, just, merciful, and God-fearing rulers in any part of the globe, are citizens of the world and united by sacred bonds of sympathy with every noble patriot upon the globe who loves and serves his country only for his country's good.

No geographical bounderies can confine the immortal names in medical science of Jenner, the discoverer of vaccine virus; of Linnaeus and Harvey, Selleman and Darlington; Crawford Long, of Georgia, who first laid the soothing hand of anæsthetics upon the body of pain. Dr. Marion Sims, of Alabama, grand oracle in surgical gynecology on two continents, and more recently, Bergeon, discoverer of sulphuretted gas to banish from so many valuable lives the dread spectre of consumption.

Their respective countries justly claim with pride the nativity of Lord Nelson, Wellington, Sir William Haverlock, Napoleon, Garabaldi, Pulaski, the brave DeKalb, Washington and Patrick Henry; but in every land all hearts that love heroism on the battle field, in national councils, or at the sick bed of a suffering brother, calls for a share in every heritage of value, exalted patriotism, and self-immolation upon the altar of duty that is left by a fellow man as a precious legacy to the world.

It is a matter of congratulation that the United States of America is at peace with all nations; and it is the devout wish of my heart that this country will never again find it needful to array itself in battle against any nation on earth. I believe that the tenderst, noblest anthem ever sung out of paradise: "Peace on earth and good will to man," is now having a greater significance to nations, and being treasured in the hearts of more individuals than ever before. God speed the day when it will be the motto emblazoned upon the crest of every sovereign on his throne, upon every nation's coat of arms, and engraven upon the hearts of every citizen and brother from the rivers to the end of the earth. How my heart would rejoice to see the dawn of that day of which England's poet laureate speaks, when "the war drum throbs no longer, and the battle flags are furled; and in the parliament of man, the grand federation of the world, all mankind will be of one heart and one mind, with one high and holy purpose in medicine, in society, in religion, in commerce and politics, and nations will embrace nation, heart to heart, soul to soul, brothers of one common country from the rising to the setting of the sun. Then the shekinah of universal love and peace will burst in glorious beauty upon the world and all the sons of earth, with one sweet and mighty song, will send back to the bending blue heavens the anthem which the angelic host chanted along the plains of Bethlehem nearly two thousands years ago, "Peace on earth and goodwill to men!"

